# Supplementary figures and images for: Population structure and antimicrobial resistance patterns of Salmonella Typhi and Paratyphi A amid a phased municipal vaccination campaign in Navi Mumbai, India
Source: mBio. 2023 Jul 28;14(4):e01179-23. doi: 10.1128/mbio.01179-23 (PMC10470601; doi:10.1128/mbio.01179-23)

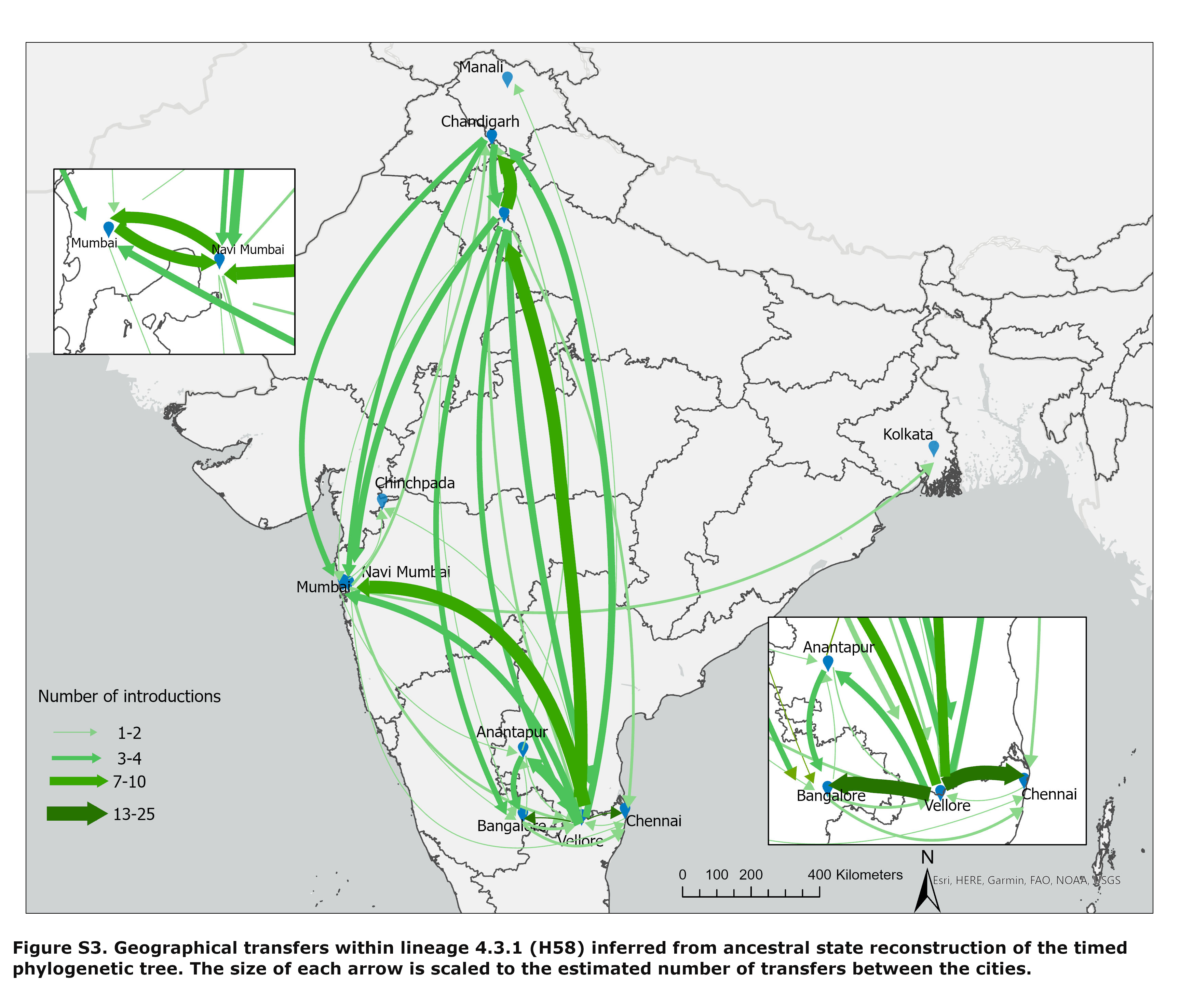

Supplement: Figure S3 — Geographical transfers within lineage 4.3.1 (H58) inferred from ancestral state reconstruction of the timed phylogenetic tree. [file mbio.01179-23-s0003.tif]
